# The association between Colombian medical students' healthy personal habits and a positive attitude toward preventive counseling: cross-sectional analyses

**DOI:** 10.1186/1471-2458-9-218

**Published:** 2009-07-03

**Authors:** John Duperly, Felipe Lobelo, Carolina Segura, Francisco Sarmiento, Deisy Herrera, Olga L Sarmiento, Erica Frank

**Affiliations:** 1 Department of Social Medicine, Universidad de los Andes Medical School, Bogotá, Colombia; 2 Department of Internal Medicine, Fundación Santa Fé, Bogotá, Colombia; 3 Department of Exercise Science, Arnold School of Public Health, University of South Carolina, Columbia, SC, USA; 4 Department of Family Practice and School of Population and Public Health, University of British Columbia, Vancouver, Canada; 5 Department of Family and Preventive Medicine, Emory University School of Medicine, Atlanta, GA, USA

## Abstract

**Background:**

Physician-delivered preventive counseling is important for the prevention and management of chronic diseases. Data from the U.S. indicates that medical students with healthy personal habits have a better attitude towards preventive counseling. However, this association and its correlates have not been addressed in rapidly urbanized settings where chronic disease prevention strategies constitute a top public health priority. This study examines the association between personal health practices and attitudes toward preventive counseling among first and fifth-year students from 8 medical schools in Bogotá, Colombia.

**Methods:**

During 2006, a total of 661 first- and fifth-year medical students completed a culturally adapted Spanish version of the "Healthy Doctor = Healthy Patient" survey (response rate = 78%). Logistic regression analyses were used to assess the association between overall personal practices on physical activity, nutrition, weight control, smoking, alcohol use (main exposure variable) and student attitudes toward preventive counseling on these issues (main outcome variable), stratified by year of training and adjusting by gender and medical training-related factors (basic knowledge, perceived adequacy of training and perception of the school's promotion on each healthy habit).

**Results:**

The median age and percentage of females for the first- and fifth-year students were 21 years and 59.5% and 25 years and 65%, respectively. After controlling for gender and medical training-related factors, consumption of ≥ 5 daily servings of fruits and/or vegetables, not being a smoker or binge drinker were associated with a positive attitude toward counseling on nutrition (*OR *= 4.71; CI = 1.6–14.1; *p *= 0.006 smoking (*OR *= 2.62; CI = 1.1–5.9; *p *= 0.022), and alcohol consumption (*OR *= 2.61; CI = 1.3–5.4; *p *= 0.009), respectively.

**Conclusion:**

As for U.S. physician and medical students, a positive association was found between the personal health habits of Colombian medical students and their corresponding attitudes toward preventive counseling, independent of gender and medial training-related factors. Our findings, the first relating to this association in medical students in developing regions, also suggest that within the medical school context, interventions focused on promoting healthy student lifestyles can potentially improve future physician's attitudes toward preventive counseling.

## Background

During 2005, non-communicable chronic diseases (NCD) accounted for over 35 million deaths worldwide [1] and projections indicate a 17% rise in the next 10 years, with more than 80% such deaths occurring in low and middle income countries [1,2]. Because of the associated health and economic burdens, national health systems must help prevent the proximal causes and underlying socioeconomic and environmental determinants of NCD [1,3].

Lifestyle modification is key to managing established NCD risk factors including physical inactivity, unhealthy diets, smoking, obesity, and alcohol use [1,4]. Moreover, physician counseling can promote behavioral changes like patient adoption and maintenance of healthy habits [5,6]. Accordingly, health care providers and particularly physicians should provide such preventive counseling and be knowledgeable on these issues [7].

Interestingly, a positive association has been found among U.S. medical students' and physicians' counseling practices and healthy personal behaviors [8,9]. Using four years of personal and clinical health-related data for a nationally representative sample of U.S. medical schools, Frank et al, found that both counseling frequency and perceived counseling relevance were positively associated with attending a school that encouraged healthy personal practices [10] and also correlated with personal health practices. Moreover, in a four-year intervention to improve medical student health at Emory University, such intervention was positively related to both personal health practices and patient counseling practices [11]. These data suggest that encouraging healthy lifestyles among medical students could facilitate the formation of healthy physicians who, in turn, would be more likely to provide preventive counseling to their patients.

Because NCD prevention can best be achieved by comprehensive public health efforts integrated with clinically relevant interventions, promoting healthy lifestyles during medical training seems a promising strategy in that the resulting physician-delivered preventive counseling could be applied at both the individual and population level. Accordingly, the few assessments of this approach's feasibility and effectiveness in the U.S. [11] must be extrapolated to other populations, especially in developing countries where NCD prevention strategies constitute a top public health priority. A first step is to understand the potentially modifiable factors associated with medical students' attitudes toward preventive counseling.

In this context, this study examined (i) the association between personal health practices and attitudes toward preventive counseling among first- and fifth-year students from 8 medical schools in Bogotá, Colombia, and (ii) the association between factors related with medical training (e.g., school environment, basic student knowledge on preventive medicine issues) and attitudes toward preventive counseling.

## Methods

### Study sample

Based on two criteria – recognition by the Colombian Association of Schools of Medicine [12] and listing in the International Medical School Directory [13]- 10 medical schools in Bogotá were invited to participate in this cross-sectional study carried out during 2006. Participation was encouraged by offering school-specific data (without student identifiers) to school investigators, relevant literature reviews and a short course on healthy lifestyle counseling to local study coordinators, and the chance to win a heart rate monitor for participating students in each school. Eight schools were selected based on a clearly expressed intention to participate and provision of the necessary conditions for survey administration. All first- and fifth-year medical students from these schools were invited to participate in a self-reported survey of personal health behaviors and attitudes toward medical practice. A total of 661 students completed the survey, a response rate of 76% for first-year and 79% for fifth-year students.

### Questionnaire

The Healthy Doctor = Healthy Patient Questionnaire, already used for a representative sample of U.S. medical schools [10,14], was translated, back-translated and culturally adapted for its use in Colombian medical schools. Dr Frank, principal investigator of the original project participated in this process to ensure questionnaire integrity and cross-country comparability. IRB approval was obtained from the Universidad de los Andes and the Fundación Santa Fe Ethical Committees.

Prior to survey administration, semi-mandatory classes and workshops explained participant anonymity, confidentiality, and the voluntary nature of participation. After the study coordinators read a 10-minute standardized script containing general instructions, the survey was administered using an electronic (Web) platform that prevented unanswered items. Completion time was on average 30 minutes.

The five sections of the questionnaire asked about the following: (i) height, weight, general health status, and family history of chronic diseases; (ii) personal habits in nutrition, physical activity, smoking, and alcohol consumption; (ii) perceptions of preventive medicine training and medical school environment; (iv) attitudes toward preventive counseling; and (v) basic knowledge (30 questions total) on smoking, alcohol use, nutrition, and physical activity. Knowledge questions in each specific area were drawn from the following sources: current recommendations for nutrition and daily energy requirements, [4,15]; health benefits and prescription of physical activity, [16]; the health effects and classification of tobacco exposure [17]; and the health effects of alcohol use and drinking status classification [15,18].

### Main outcome measures

Outcome measures and independent variables were selected based on previous investigations of correlates of medical students' prevention counseling practices [10] and the specifics of medical training and practice in Colombia and the Latin-American region [19].

Our primary outcome, student attitudes toward preventive counseling on healthy lifestyles, was assessed by asking "how relevant do you think this will be in your intended practice – talking to patients about physical activity, nutrition, smoking, alcohol use, weight management". Students reporting "highly" were considered to have a positive attitude toward always counseling patients on each specific habit; those reporting "somewhat" or "not at all" were classified as not having a positive attitude toward preventive counseling.

### Independent variables

Student reports of personal habits were considered the primary independent variables. Compliance with international physical activity recommendations (≥ 150 min/week of moderate-to-vigorous physical activity) [20] was assessed by inquiring the average duration of vigorous and moderate physical activities in the past week. Compliance with international recommendations for fruit and/or vegetable consumption (≥ 5 servings per day) [21] was assessed using a validated food frequency tool. Students reporting current use of tobacco and having smoked ≥ 100 cigarettes during their lives were considered smokers [22]. Students reporting consumption of ≥ 5 drinks of alcohol on a single occasion in the past month were considered binge drinkers. Women reporting an average consumption of >1 daily drink or men an average of >2 daily drinks in the past month were considered heavy drinkers [15,18] Students meeting physical activity, and fruit and vegetable recommendations, who were also non-smokers and neither heavy nor binge drinkers were considered to have overall healthy habits.

Covariates included other factors associated with medical training. Students were designated to have a perception that their medical school encourages healthy behaviors on each individual habit and promotes health in this area ("My medical school encourages their students to be physically active, to eat healthily and discourages smoking and alcohol drinking"), and to have a perception that they received adequate training on this area were based on responses to the question ("How much training have you had on the topics of nutrition, exercise/physical activity, smoking cessation and alcohol?"). Adequate knowledge on each area (physical activity, nutrition, alcohol consumption and smoking) was defined as having at least 60% correct answers on each group of questions in order to objectively assess students' basic knowledge and competency on each habit.

### Statistical analysis

We anticipated *a priori *that student understanding of and attitude toward counseling and medical training-related factors could be affected by clinical experience and time in the medical school environment, respectively. Because the two groups (first- and fifth-year students) were very distinct, analysis of these variables was stratified by year of training (no data were pooled). After first estimating the prevalence of reported healthy habits, weight status, attitude toward preventive counseling and perception of school environment, we compared the prevalence estimates for the two groups using chi-square tests. Second, we used logistic regression analysis to assess the association between overall healthy habits (physical activity, nutrition, weight control, smoking, alcohol use) and student attitudes toward preventive counseling on these issues, stratified by year of training. Third, to assess effect modification, we further stratified the analysis, only among fifth-year students, by medical training-related factors, including basic knowledge about each healthy habit, perception of the school's promotion of each healthy habit, and perceived adequacy of training on each healthy habit. Finally, we used multivariate models, adjusted for gender and the medical training-related factors, to assess the association between fifth-year students' personal practices on each habit and their attitudes toward preventive counseling. Collinearity between independent variables was evaluated using regression diagnostic tests. All analyses were conducted using SAS v 9.1[23].

## Results

### Study population

The median age and percentage of females for the first and fifth-year students were 21 years and 59.5% and 25 years and 65% respectively, with over 93% of students reporting middle and high socioeconomic status. For both groups, we found low compliance with the recommendations on fruit and vegetable consumption and physical activity, and a high prevalence of binge drinking (Table 1). Compared to first-year students, the proportion of non-smokers and non-binge drinkers/abstainers was lower among fifth-year students (83% vs. 75%; *p *= 0.023 and 57% vs. 42%; *p *= 0.002, respectively). Most students (≥ 78%) in both years had a favorable attitude toward preventive counseling on individual habits, but the proportion of positive attitude toward physical activity counseling was higher for fifth- than first-year students (91% vs. 85%; *p *= 0.016). Additionally, the proportion of students perceiving school promotion of each healthy habit was lower among fifth- than among first-year students for nutrition, physical activity, and alcohol consumption.

**Table 1:** Personal habits, attitude toward preventive counseling, and perception of school environment among Colombian medical students

	1st year (*n *= 407)	5th year (*n *= 254)	
Characteristics	%	%	*p*-value
Body mass index (Kg/m^2^)			
<18.5	11	9	0.16
18.5 – 24.9	80	78	0.64
25 – 30	8	11	0.09
>30	1	2	0.16
			
Students' reported healthy habits			
Consuming ≥ 5 daily servings of fruits and/or vegetables	41	40	0.80
			
≥ 150 min/week of moderate-to-vigorous physical activity	51	50	0.73
Non-smoking^a^	83	75	0.02
Non-binge drinking^b^	57	42	0.002
Non-heavy drinking^c^	88	82	0.08
Overall healthy habits^d^	12	8	0.13
			
Students with a positive attitude^e ^toward always counseling patients about:			
Nutrition	93	89	0.06
Physical activity	85	91	0.02
Smoking	89	89	0.90
Alcohol consumption	78	80	0.51
Weight control	90	94	0.09
All healthy habits^f^	68	73	0.19
			
Students' perception of the school environment as promoting each healthy habit:			
Nutrition	43	22	<0.0001
Physical activity	46	30	<0.0001
Smoking	43	47	0.36
Alcohol consumption	42	28	0.0003

^a ^Smokers: Students reporting current use of tobacco and having smoked ≥ 100 cigarettes during their lives, ^b ^Binge drinkers: those who consume ≥ 5 drinks of alcohol on a single occasion, ^c ^Heavy drinkers: women who consume an average of >1 daily drink or men consuming an average of >2 daily drinks, ^d ^Students with overall healthy habits: those who report doing ≥ 150 minutes a week of moderate-to-vigorous physical activity, consuming ≥ 5 servings of fruit and/or vegetables a day, and being nonsmokers and non-heavy or non-binge drinkers, ^e ^Positive attitude toward counseling: students that agreed or strongly agreed that if is important for physicians to counsel patients about each individual habit, ^f ^All healthy habits: nutrition, physical activity, smoking, alcohol consumption, weight control.

*Note, p*-value: chi-square for 1st vs. 5th year differences.

### Personal habits and attitude toward preventive counseling on healthy habits

Among first-year students, non-binge drinkers were more likely to have a positive attitude toward counseling patients about alcohol use (*OR *1.79; 95% CI 1.12–2.87) than binge drinkers (Additional file 1). Among fifth-year students, we found positive associations between compliance with nutritional recommendations, being a non-smoker, and not being a binge-drinker with a positive attitude toward counseling on nutrition, smoking and alcohol use, respectively. In addition, fifth-year students complying with the nutritional recommendation were much more likely to have a positive attitude toward counseling patients about weight control (*OR *10.25; 95% CI 1.13–79.19) (Additional file 1). We found no evidence that medical training-related variables for each health-related habit (basic knowledge, student perception of school's promotion of a healthy environment, perception of adequate training) acted as effect modifiers of the association between fifth-year students personal habits and attitude toward counseling on each corresponding healthy habit (data not shown).

Finally, the multivariate logistic regression analyses showed that after controlling for gender and medical training-related factors, consumption of ≥ 5 daily servings of fruits and/or vegetables, not being a smoker or binge drinker were associated with a positive attitude toward counseling on nutrition (*OR *= 4.71; CI = 1.6–14.1; *p *= 0.006), smoking (*OR *= 2.62; CI = 1.1–5.9; *p *= 0.022), and alcohol consumption (*OR *= 2.61; CI = 1.3–5.4; *p *= 0.009), respectively (Additional file 2).

## Discussion

Overall, we found a positive association among Colombian medical students, between reported healthy personal habits in nutrition, smoking and alcohol consumption and a favorable attitude toward counseling on each corresponding habit. Although detected consistently in senior but not freshman medical students, this association was still independent of medical training-related factors including the students' basic knowledge of and perceived training adequacy on healthy lifestyles, and their perception of a school environment that promotes healthy habits. Such associations between personal practices and attitudes toward (and practice of) preventive counseling have been apparent among medical students and practicing physicians in the U.S. [10] but to our knowledge, has not been studied elsewhere. Collectively, the results of this and previous investigations suggest that improving students' personal habits during medical training may have a beneficial impact on future physicians' counseling attitudes.

The association between medical students' personal habits and specific counseling attitudes can be linked to the premises of behavioral models [24]. That is, lifestyle habits can be transferred into professional practice – in this case, preventive counseling – where physicians' discussion of personal healthy eating or active lifestyle practices makes the message more credible, coherent, and motivating for patients [6]. However, as shown here and elsewhere, favorable attitudes toward preventive counseling and better health promotion practices are more likely to be found in healthier students [10,25]; therefore, healthy habit promotion at the medical school level could improve patient counseling practices [11]. Accordingly, evidence-based behavioral change strategies could be implemented in medical school training to improve future doctors' health habits and attitudes toward preventive counseling.

Compared with other young Colombian adults, the medical students surveyed here reported good habits for some but not all health behaviors. On the positive side, compliance with physical activity was higher and prevalence of overweight lower among medical students when compared to the general age-matched Colombian population (50.5% vs. 40.4% and 10.4% vs. 18%, respectively). [26]. However, compared to the 19–50 year-old Colombian population, a lower proportion of medical students reported consuming at least one vegetable (74% vs. 53%) and one fruit per day (64.2% vs. 61%), respectively (data not shown) [26]. Additionally, the prevalence of smoking was also higher among the medical students than the general population aged 18–24 (20.3% vs. 14.7%) [27]. These figures that are in line with a previous report of a smoking prevalence of 25.9% among 2,021 first and fifth-year students from 11 medical schools across Colombia [28] but are in contrast to lower smoking rates among medical students and physicians in the U.S. [29]. Overall, the data indicate that Colombian medical students exhibit some healthier behaviors than their age-matched peers elsewhere, including a higher compliance with physical activity recommendations and a lower prevalence of unhealthy weight. However, significant room exists for improvement, especially in smoking and fruit and vegetable consumption habits.

In this study, senior students reporting healthy personal habits were more likely to have a positive attitude toward counseling on each corresponding habit, with 4 out of 6 *a priori *associations being significant (e.g. consuming ≥ 5 daily servings of fruits and/or vegetables). These results show remarkable consistency with findings for U.S. medical students, including a significant correlation between a personal health index (smoking, drinking, exercise, and diet habits) and perceived relevance of prevention counseling in the intended practice [10] and associations between perceived relevance of nutrition or exercise counseling and consuming more fruits and vegetables [30] or complying with physical activity recommendations [14], respectively.

Our analysis also took into consideration the earlier finding that among U.S. students, the school's health promotion score (*p *= 0.0007), female gender (*p *< 0.0001), and non-White ethnicity (*p *< 0.0001) or intention to specialize in non primary care (*p *< 0.0001) were predictors of the perceived relevance of preventive counseling [10].

Among Colombian senior students, we found that once gender, student perception of the school's health promotion efforts, basic knowledge and perception of training adequacy for each healthy habit were accounted for, healthy practices remained associated with a positive attitude toward preventive counseling for nutrition, physical activity, smoking, and alcohol use. However, the fact that this association between personal habits and corresponding preventive counseling was consistently detected among fifth- but not first-year medical students indicates that this relationship probably develops during the years of medical training.

### Study limitations and strengths

The study limitations include its cross-sectional nature and the possibility of self-report biases; however, the questions related to lifestyle habits were extracted from recognized epidemiological surveillance tools [31], which in turn enabled comparisons with national estimates for these habits. However, since the study was limited to students attending mostly private medical schools in Bogotá, extrapolation of these results to students attending public schools or in other regions should be performed cautiously.

The study's strengths include a high response rate, the availability of objective data on knowledge about healthy habits, and the use of the extensively tested Healthy Doctor = Healthy Patient questionnaire whose primary outcome measure of self-reported counseling rates has been objectively validated [32]. Future research including intervention studies in developing countries that demonstrate the effectiveness of healthy behavior promotion among medical students for improving attitudes towards and delivery of preventive counseling are warranted and will help bridge the gap between medical students' behaviors and related attitudes towards counseling and physicians' preventive practices.

## Conclusion

Our study is the first to show a positive association between the personal health habits of Colombian medical students and their corresponding attitudes toward preventive counseling and to demonstrate that, despite differences in health behaviors, the Healthy Doctor = Healthy Patient association is consistent among both Colombian and U.S. medical students. Our findings, the first relating to this association in medical students in developing regions, also suggest that within the medical school context, interventions focused on promoting healthy student lifestyles can potentially improve future physicians' attitudes toward preventive counseling.

## Competing interests

The authors declare that they have no competing interests.

## Authors' contributions

JD, FL, CS, OLS and EF were responsible for conception of the study, study design, set up and data collection and processing. CS, FS and DE coordinated the data collection and processing. FL and OLS analyzed the data. JD and FL drafted the manuscript. All authors were involved with data interpretation, critical revisions of the paper and provided approval for its publication.

## Pre-publication history

The pre-publication history for this paper can be accessed here:



## Supplementary Material

Additional file 1 **Association between habits and attitude towards counseling on healthy habits among Colombian medical students.** Click here for file

Additional file 2 **Models of a positive attitude toward prevention counseling^a ^among Colombian medical students.** Click here for file
