# Supplementary material for: The association between Colombian medical students' healthy personal habits and a positive attitude toward preventive counseling: cross-sectional analyses
Source: BMC Public Health. 2009 Jul 3;9:218. doi: 10.1186/1471-2458-9-218 (PMC2721845; doi:10.1186/1471-2458-9-218)
Supplement: Additional file 1 — Association between habits and attitude towards counseling on healthy habits among Colombian medical students. [file 1471-2458-9-218-S1.doc]

| Table 2. Association between habits and attitude towards counseling on healthy habits among Colombian medical students | | | | | | | | | | | | |
| --- | --- | --- | --- | --- | --- | --- | --- | --- | --- | --- | --- | --- |
|  | Students with a positive attitude^a^ toward always counseling patients about | | | | | | | | | | | |
| Student´s personal habits | Nutrition | | Physical activity | | Smoking | | Alcohol consumption | | Weight control | | All  healthy habits^b^ | |
|  | OR | (95% CI) | OR | (95% CI) | OR | (95% CI) | OR | (95% CI) | OR | (95% CI) | OR | (95% CI) |
| *Total students* (n = 661) |  |  |  |  |  |  |  |  |  |  |  |  |
| Not consuming ≥5 daily servings of fruits  and/or vegetables (referent) | 1.00 | - | 1.00 | - | 1.00 | - | 1.00 | - | 1.00 | - | 1.00 | - |
| Consuming ≥5 daily  servings of fruits and/or vegetables | ***2.30*** | ***(1.23-4.29)*** | 1.81 | (1.09-2.99) | 1.01 | (0.61-1.65) | 1.32 | (0.90-1.95) | 1.70 | (0.92-3.11) | **1.61** | **(1.14-2.29)** |
| Not engaging in ≥150 min/week of moderate-  to-vigorous physical activity (referent) | 1.00 | - | 1.00 | - | 1.00 | - | 1.00 | - | 1.00 | - | 1.00 | - |
| Engaging in ≥150 min/week  of moderate-to-vigorous physical activity | 0.98 | (0.56-1.73) | 1.40 | (0.88-2.25) | 0.96 | (0.58-1.59) | 1.05 | (0.72-1.53) | 0.91 | (0.52-1.60) | 1.01 | (0.72-1.42) |
| Smoking (referent) | 1.00 | - | 1.00 | - | 1.00 | - | 1.00 | - | 1.00 | - | 1.00 | - |
| No smoking^c^ | 0.60 | (0.28-1.31) | 0.50 | (0.25-1.00) | **1.79** | **(1.04-3.06)** | 1.08 | (0.68-1.69) | 0.29 | (0.10-0.82) | 1.13 | (0.75-1.70) |
| Binge drinking (referent) | 1.00 | - | 1.00 | - | 1.00 | - | 1.00 | - | 1.00 | - | 1.00 | - |
| No binge drinking^d^ | 1.53 | (0.89-2.64) | 1.27 | (0.80-2.01) | 1.51 | (0.93-2.47) | **2.00** | **(1.36-2.93)** | 0.75 | (0.43-1.32) | 1.82 | (1.30-2.55) |
| *1st year* (n = 407) |  |  |  |  |  |  |  |  |  |  |  |  |
| Not consuming ≥5 daily servings of fruits  and/or vegetables (referent) | 1.00 | - | 1.00 | - | 1.00 | - | 1.00 | - | 1.00 | - | 1.00 | - |
| Consuming ≥5 daily  servings of fruits and/or vegetables | *1.35* | *(0.61-2.98)* | 1.51 | (0.85-2.69) | 0.81 | (0.44-1.50) | 1.12 | (0.69-1.81) | *1.12* | *(0.57-2.21)* | 1.40 | (0.91-2.15) |
| Not engaging in ≥150 min/week of moderate-  to-vigorous physical activity (referent) | 1.00 | - | 1.00 | - | 1.00 | - | 1.00 | - | 1.00 | - | 1.00 | - |
| Engaging in ≥150 min/week  of moderate-to-vigorous physical activity | 0.97 | (0.44-2.12) | *1.34* | *(0.77-2.33)* | 0.90 | (0.48-1.70) | 0.92 | (0.57-1.47) | *0.70* | *(0.36-1.38)* | 0.94 | (0.62-1.44) |
| Smoking (referent) | 1.00 | - | 1.00 | - | 1.00 | - | 1.00 | - | 1.00 | - | 1.00 | - |
| No smoking | 0.52 | (0.15-1.78) | 0.67 | (0.30-1.49) | *1.36* | *(0.64-2.89)* | 0.75 | (0.39-1.44) | 0.37 | (0.11-1.22) | 0.96 | (0.55-1.67) |
| Binge drinking (referent) | 1.00 | - | 1.00 | - | 1.00 | - | 1.00 | - | 1.00 | - | 1.00 | - |
| No binge drinking | 1.25 | (0.59-2.66) | 1.55 | (0.90-2.68) | 1.66 | (0.90-3.07) | ***1.79*** | ***(1.12-2.87)*** | 0.71 | (0.36-1.42) | **1.89** | **(1.24-2.88)** |
|  |  |  |  |  |  |  |  |  |  |  |  |  |
| *5th year* (n = 254) |  |  |  |  |  |  |  |  |  |  |  |  |
| Not consuming ≥5 daily servings of fruits  and/or vegetables (referent) | 1.00 | - | 1.00 | - | 1.00 | - | 1.00 | - | 1.00 | - | 1.00 | - |
| Consuming ≥5 daily  servings of fruits and/or vegetables | ***4.82*** | ***(1.63-14.31)*** | **3.29** | **(1.08-10.02)** | 1.48 | (0.64-3.41) | 1.80 | (0.93-3.49) | ***10.25*** | ***(1.33-79.19)*** | **2.14** | **(1.17-3.91)** |
| Not engaging in ≥150 min/week of moderate-  to-vigorous physical activity (referent) | 1.00 | - | 1.00 | - | 1.00 | - | 1.00 | - | 1.00 | - | 1.00 | - |
| Engaging in ≥150 min/week  of moderate-to-vigorous physical activity | 0.98 | (0.43-2.22) | *1.67* | *(0.67-4.19)* | 1.07 | (0.47-2.46) | 1.34 | (0.71-2.54) | *1.83* | *(0.60-5.64)* | 1.16 | (0.65-2.06) |
| Smoking (referent) | 1.00 | - | 1.00 | - | 1.00 | - | 1.00 | - | 1.00 | - | 1.00 | - |
| No smoking | 0.60 | (0.22-1.64) | 0.28 | (0.06-1.23) | ***2.57*** | ***(1.14-5.79)*** | 1.71 | (0.87-3.33) | 0.20 | (0.03-1.58) | 1.49 | (0.80-2.76) |
| Binge drinking (referent) | 1.00 | - | 1.00 | - | 1.00 | - | 1.00 | - | 1.00 | - | 1.00 | - |
| No binge drinking | 1.68 | (0.73-3.86) | 1.04 | (0.43-2.52) | 1.33 | (0.59-3.01) | ***2.80*** | ***(1.39-5.66)*** | 1.08 | (0.37-3.13) | **1.94** | **(1.08-3.50)** |
| ^a^ Positive attitude towards counseling: students that agreed or strongly agreed that it is important for physicians to counsel patients about each individual habit, ^b^ All healthy habits: nutrition, physical activity, smoking, alcohol consumption, and weight control, ^c^ Smokers: reporting current use of tobacco or having smoked ≥100 cigarettes during their lives , ^d^ Binge drinkers: those who consume ≥5 drinks of alcohol on a single occasion, ^d^ overall healthy habits: those who report doing ≥150 minutes a week of moderate physical activity, consuming ≥5 servings of fruit and/or vegetables a day, and being non-smokers and non-heavy or non-binge drinkers. *Note:* Hypothesis-driven associations are in *italics*. **Statistically significant associations are in bold.** | | | | | | | | | | | | |
