# Supplementary material for: The association between Colombian medical students' healthy personal habits and a positive attitude toward preventive counseling: cross-sectional analyses
Source: BMC Public Health. 2009 Jul 3;9:218. doi: 10.1186/1471-2458-9-218 (PMC2721845; doi:10.1186/1471-2458-9-218)
Supplement: Additional file 2 — Models of a positive attitude toward prevention counselinga among Colombian medical students. [file 1471-2458-9-218-S2.doc]

| Table 3. Models of a positive attitude toward prevention counseling^a^ among Colombian medical students | | | |
| --- | --- | --- | --- |
|  | OR | (95% CI) | *p-* value |
| Nutrition |  |  |  |
| Gender = female (referent) | 1.00 | - | - |
| Gender = male | 0.81 | 0.4-1.8 | 0.628 |
| Reports NOT consuming ≥5 daily servings of fruits and/or vegetables (referent) | 1.00 | - | - |
| **Reports consuming ≥5 daily servings of fruits and/or vegetables** | **4.71** | **1.6-14.1** | **0.006** |
| Insufficient basic knowledge about healthy nutrition (referent) | 1.00 | - | - |
| Sufficient basic knowledge^b^ about healthy nutrition | 1.03 | 0.3-3.2 | 0.957 |
| Perception of a negative school environment promoting^c^ healthy nutrition (referent) | 1.00 | - | - |
| Perception of a positive school environment promoting^c^ healthy nutrition | 1.20 | 0.4-3.5 | 0.738 |
| Perception of inadequate training^d^ on nutrition (referent) | 1.00 | - | - |
| Perception of adequate training^d^ on nutrition | 0.78 | 0.3-1.9 | 0.57 |
|  |  |  |  |
| Physical activity |  |  |  |
| Gender = female (referent) | 1.00 | - | - |
| Gender = male | 1.07 | 0.4-2.8 | 0.886 |
| Engages in ≥150 min/week of moderate-to-vigorous physical activity (referent) | 1.00 | - | - |
| Engages in ≥150 min/week of moderate-to-vigorous physical activity | 1.73 | 0.6-4.4 | 0.25 |
| Insufficient basic knowledge about physical activity (referent) | 1.00 | - | - |
| Sufficient basic knowledge about physical activity | 0.71 | 0.2-1.9 | 0.512 |
| Perception of a negative school environment promoting physical activity (referent) | 1.00 | - | - |
| Perception of a positive school environment promoting physical activity | 1.12 | 0.4-3.1 | 0.833 |
| Perception of inadequate training on physical activity (referent) | 1.00 | - | - |
| Perception of adequate training on physical activity | 0.94 | 0.4-2.4 | 0.894 |
|  |  |  |  |
| Smoking |  |  |  |
| Gender = female (referent) | 1.00 | - | - |
| Gender = male | 0.69 | 0.3-1.5 | 0.376 |
| Smoking (referent) | 1.00 | - | - |
| **No smoking** | **2.62** | **1.1-5.9** | **0.022** |
| Sufficient basic knowledge about smoking (referent) | 1.00 | - | - |
| Insufficient basic knowledge about smoking | 1.66 | 0.7-3.7 | 0.232 |
| Perception of a negative school environment that discourages smoking (referent) | 1.00 | - | - |
| Perception of a positive school environment that discourages smoking | 1.73 | 0.7-4.1 | 0.221 |
| Perception of inadequate training on smoking (referent) | 1.00 | - | - |
| Perception of adequate training on smoking | 1.15 | 0.5-2.7 | 0.759 |
|  |  |  |  |
| Alcohol consumption |  |  |  |
| Gender = female (referent) | 1.00 | - | - |
| Gender = male | 0.66 | 0.3-1.3 | 0.215 |
| Binge drinking | 1.00 | - | - |
| **No binge drinking** | **2.61** | **1.3-5.4** | **0.009** |
| Insufficient basic knowledge about alcohol consumption | 1.00 | - | - |
| Sufficient basic knowledge about alcohol consumption | 1.1 | 0.5-2.5 | 0.822 |
| Perception of a negative school environment that discourages alcohol consumption (referent) | 1.00 | - | - |
| Perception of a positive school environment that discourages alcohol consumption | 1.25 | 0.6-2.6 | 0.557 |
| Perception of inadequate training on alcohol consumption (referent) | 1.00 | - | - |
| Perception of adequate training on alcohol consumption | 1.37 | 0.7-2.6 | 0.339 |
| ^a^ Positive attitude towards counseling: students that agreed or strongly agreed that is it important for physicians to counsel patients about each healthy habit, ^b^ Having answered correctly at least 60% of the basic knowledge questions on each topic, ^c^ Students that agreed or strongly agreed their medical school has an adequate environment to promote each healthy habit, ^d^ Students that agreed or strongly agreed that their medical school gives them adequate training on each healthy habit. *Note:* Significant factors are in bold. | | | |
